# Ultrasensitive Detection of Multidrug-Resistant *Mycobacterium tuberculosis* Using SuperSelective Primer-Based Real-Time PCR Assays

**DOI:** 10.3390/ijms232415752

**Published:** 2022-12-12

**Authors:** Anshika Narang, Salvatore A. E. Marras, Natalia Kurepina, Varsha Chauhan, Elena Shashkina, Barry Kreiswirth, Mandira Varma-Basil, Christopher Vinnard, Selvakumar Subbian

**Affiliations:** 1 Public Health Research Institute, New Jersey Medical School, Rutgers University, Newark, NJ 07103, USA; 2 Center for Discovery and Innovation, Nutley, NJ 07110, USA; 3 Department of Microbiology, Vallabhbhai Patel Chest Institute, University of Delhi, Delhi 110021, India; 4 New Jersey Medical School, Rutgers University, Newark, NJ 07103, USA

**Keywords:** drug resistance, molecular diagnosis, anti-tuberculosis therapy, Real-Time PCR, isoniazid, rifampicin, genomic DNA, mutation

## Abstract

The emergence of drug-resistant tuberculosis is a significant global health issue. The presence of heteroresistant *Mycobacterium tuberculosis* is critical to developing fully drug-resistant tuberculosis cases. The currently available molecular techniques may detect one copy of mutant bacterial genomic DNA in the presence of about 1–1000 copies of wild-type *M. tuberculosis* DNA. To improve the limit of heteroresistance detection, we developed SuperSelective primer-based real-time PCR assays, which, by their unique assay design, enable selective and exponential amplification of selected point mutations in the presence of abundant wild-type DNA. We designed SuperSelective primers to detect genetic mutations associated with *M. tuberculosis* resistance to the anti-tuberculosis drugs isoniazid and rifampin. We evaluated the efficiency of our assay in detecting heteroresistant *M. tuberculosis* strains using genomic DNA isolated from laboratory strains and clinical isolates from the sputum of tuberculosis patients. Results show that our assays detected heteroresistant mutations with a specificity of 100% in a background of up to 10^4^ copies of wild-type *M. tuberculosis* genomic DNA, corresponding to a detection limit of 0.01%. Therefore, the SuperSelective primer-based RT-PCR assay is an ultrasensitive tool that can efficiently diagnose heteroresistant tuberculosis in clinical specimens and contributes to understanding the drug resistance mechanisms. This approach can improve the management of antimicrobial resistance in tuberculosis and other infectious diseases.

## 1. Introduction

Tuberculosis (TB) is currently the second leading infectious killer disease worldwide after COVID-19, with a disease burden shared across all countries, genders, and age groups. In 2020, an estimated 10 million people fell ill with TB, and 1.5 million people died [1]. Multidrug-resistant TB (MDR-TB), defined as resistance to isoniazid (INH) and rifampin (RIF), remains a serious public health threat worldwide. Preventing the emergence of bacterial drug resistance is a critical component of TB elimination efforts, with the World Health Organization (WHO) aiming to reduce 95% of TB-attributable deaths by 2035 [1].

The standard regimen of directly observed therapy short course (DOTS) strategy for global TB control includes 4 months of treatment with INH, RIF, pyrazinamide (PZA), and ethambutol (EMB), followed by 2 months of INH and RIF [1]. Single-nucleotide polymorphisms (SNPs) at a handful of genetic loci (known as hot spots) are responsible for the development of the majority of drug resistance in *M. tuberculosis* [2]. MDR-TB is thought to develop first with the selection of INH-resistant mutant *M. tuberculosis* strains, followed by the selection of RIF resistance among these mutants [3]. Treatment of MDR-TB requires the use of more toxic, more expensive, and less effective second-line drugs administered for a prolonged time (more than a year) [4], although shorter MDR treatment regimens have been endorsed by WHO [5].

Acquired drug resistance in TB occurs via the selection of spontaneous mutants in a wild-type (WT) background with a spontaneous mutation rate of 10^−8^ to 10^−9^ per round of *M. tuberculosis* replication [6]. With a sufficiently large *M. tuberculosis* population in infected tissue (greater than 10^9^ organisms) [7], spontaneous mutants with resistance to a single drug are likely to be present, which could then be selected by inadequate drug pressure [3]. Heteroresistant TB is defined as a disease in which drug-resistant and drug-susceptible *M. tuberculosis* strains co-exist in the same patient [8,9,10]. In the conventional proportional drug sensitivity tests (pDST), heteroresistance is defined as 1–99% bacterial colony growth on drug-containing media [11].

Current TB diagnostic methods are insensitive in detecting low levels of mutant *M. tuberculosis* in a WT background, with lower limits of quantification ranging between 1 and 10% for standard methods, such as line probe assays [12,13] and mycobacteria growth indicator tubes (MGIT) [14]. Critically, the major limitation of traditional polymerase chain reaction (PCR) methodology is the inability to detect rare mutant sequences in a background of abundant WT DNA. With recent advances, such as digital PCR [10] and next-generation sequencing [15], the limit of detection of rare mutants may approach the 0.1% threshold (corresponding to the ratio of mutant DNA/WT DNA), which remains far above the level required to detect the baseline presence of heteroresistant strains in clinical samples or dynamic changes in the emergence of drug-resistant *M. tuberculosis* [7]. Even with the limitations of current tools, the clinical impact of heteroresistant TB is increasingly recognized [8,11,14,16]. A recent study reported the weighted pooled prevalence of INH heteroresistance (from 19 studies) to be 5% and RIF heteroresistance (from 17 studies) to be 7% from 2001 to 2020 [17]. Among South African TB patients, heteroresistant infections identified by the mycobacterial interspersed repetitive units variable number of tandem repeat (MIRU-VNTR) genotyping were associated with a 90% increase in the odds of delayed sputum conversion after 2 months of treatment [18], a critical treatment endpoint for TB control programs (and related to the risk of ongoing transmission in the community). Heteroresistant *M. tuberculosis* infection can lead to a false negative rapid nucleic acid test for RIF resistance [19,20] and contributes to variable treatment responses observed in different anatomic regions of infected lungs [21]. Most worrisome, patients with clinically undetected heteroresistance can progress to MDR-TB in the face of first-line treatment pressure [22], either due to formulation differences [23] or intra-individual pharmacokinetic variability [24]. All of these mechanisms contribute to the failure of TB control efforts through inadequate treatment and the further spread of drug-resistant *M. tuberculosis* strains [3].

In this report, we propose a revolutionary new approach in PCR primer design, called “SuperSelective PCR primers” [25], for ultrasensitive detection of drug-resistant *M. tuberculosis* bacilli in an abundant background of wild-type *M. tuberculosis*. Our innovation in PCR primer design and demonstration of these primers in RT-PCR assays provide a significant technological advancement with clinical application toward detecting rare drug-resistant mutants in TB and other infectious diseases. SuperSelective primers innovate by separating a relatively long “anchor” sequence targeting the gene of interest- and a short “foot” sequence selective for the point mutation (i.e., the interrogating nucleotide). In between, there is a “bridge” sequence, which is non-binding to the target DNA sequence. When the SuperSelective primer is hybridized to the template molecule, the bridge sequence in the primer and the corresponding template sequence make a “bubble” region that functionally separates the efficient formation of the anchor hybrid with the formation of the foot hybrid [25]. Both the anchor (corresponding to the target gene) and foot (with the interrogating nucleotide) sequences must bind to the DNA fragment for amplification to proceed. Use of SuperSelective PCR primers in RT-PCR overcomes the limitations of other primer-based approaches, like amplification refractory mutation system (ARMS) primers [26], dual-priming oligonucleotide (DPO) primers [27], hairpin primers [28], and PlexPrimers [29], which are either not sufficiently sensitive to detect extremely rare mutants, not compatible with real-time PCR due to the presence of unnatural nucleotides in their sequence, or have not been shown to enable quantitative determinations in multiplex real-time PCR assays when different target mutations occur in the same codon. By relying on standard PCR equipment and reagents, SuperSelective PCR methods can be readily adopted by any laboratory equipped to perform conventional PCR, supporting implementation in settings with medium and high burdens of TB disease.

## 2. Results

### 2.1. SuperSelective Primer Design

We first designed a prototype SuperSelective primer to detect *inhA* promoter mutation -15C→T and optimized the primer configuration and PCR conditions to achieve maximum sensitivity for mutant detection. A SuperSelective primer 18-14/14-6:1:0 (inh15_SSP3) was paired with a conventional reverse primer and tested in a RT-PCR assay to detect one copy of -15C→T mutant in a background of 10^5^ copies of WT DNA. Linearized pUC plasmid containing a cloned sequence carrying *inhA* promoter mutation -15C→T was used as a template for these experiments, and its WT counterpart was used as background. Results show that our primer design efficiently amplifies as low as one copy of mutant DNA (in three out of six replicates) in a background of 10^5^ copies of WT DNA. Amplification of mutant in the control set of the experiment (10^5^ copies of mutant DNA without the WT background) down to one copy confirmed that the amplification signal for mutant from low copy numbers was specific and not a product of the non-specific binding of primers to the background WT pDNA. Although this SuperSelective primer design also resulted in the amplification of WT DNA (one out of six replicates), the WT amplification peaks were seen occasionally at PCR cycle (Ct) > 40, which overlapped with the amplification peaks with low copy numbers (ten and one) of mutant. The non-suppression of the unintended WT target amplification using this SuperSelective primer could be attributed to the high GC content in *M. tuberculosis* gDNA [30], leading to a firm binding of anchor and/or foot regions to the WT template.

The main objective of our investigation was to design primers adequate for early amplification of mutant (ideally Ct 19–25 for 10^5^ copies) to attain amplification down to 1 copy within a short RT-PCR cycle (ideally 50 cycles), with complete suppression of WT background. Therefore, to determine optimal parameters for this assay, we tweaked the SuperSelective primer design in different parts of the PCR (owing to different functions), making one to two variations at a time, and tested these modified designs under different annealing temperatures (Tm) and assay conditions (Table S1).

### 2.2. Standardization of Primer Foot Length

To strengthen the binding of SuperSelective primers with the mutant DNA template, we tested SuperSelective primer 18-14/13-7:1:0 (inh15_SSP4) with a longer foot length (8 nt instead of 7 nt) in the RT-PCR assay described as above. The resulting Ct values of 10^5^ copies of mutant and 10^5^ copies of WT DNA with this longer foot primer were plotted against the Ct values obtained with primer 18-14/14-6:1:0 (inh15_SSP3). Results show that the extended foot length (8 bp) in primer inh15_SSP4 helped in the early amplification of mutant DNA template (3–4 cycles early) but also enhanced the binding of SuperSelective primers with WT DNA (Figure S1a). These results demonstrate that a shorter foot length may delay achieving the threshold cycle, leading to enhanced selectivity, whereas a longer foot length may reduce the selectivity of SuperSelective primers. From a thermodynamic standpoint, the improved selectivity with shorter foot lengths can be due to the higher ratio of the equilibrium abundance of perfectly complementary mutant foot hybrids compared to the equilibrium abundance of mismatched WT foot hybrids [25].

### 2.3. Standardization of the Size and Symmetry of Intervening Loop (Bubble)

In order to inhibit the amplification of WT gene copy or delay the amplification beyond 50 cycles, which would avoid overlap with amplification of low copies of mutant, we tested SuperSelective primers 18-14/21-6:1:0 (inh15_SSP2) with increased lengths of the intervening sequence (from 14 to 21 bp) (forming an asymmetric bubble) in RT-PCR assays. The resulting Ct values of 10^5^ copies of mutant and 10^5^ copies of WT with this primer were plotted against the Ct values obtained with 18-14/14-6:1:0 (inh15_SSP3), which has a shorter length of the intervening sequence (equal to the length of the bridge, forming a symmetric bubble) (Figure S1b). Two reactions were carried out with each of the primers, of which 18-14/21-6:1:0 initiated the amplification of WT in one reaction and completely suppressed the amplification in the other sample. In contrast, the 18-14/14-6:1:0 primer amplified the targets of both WT replicate reactions. The asymmetric bubble with 18-14/21-6:1:0 also resulted in a Ct delay (~5 cycles) in the amplification of the mutant target and lower fluorescence signal. These results signify that the asymmetricity of the bubble, created by the intervening sequence, may significantly affect the binding efficiency of a SuperSelective primer with its target and, thus, the amplification threshold, resulting in the formation of fewer amplicons.

We also tested SuperSelective primers with a variation (15/17 nt) in bridge length of the primer design 18-14/13-7:1:0 (inh15_SSP4). In all other aspects, the composition of the primers was the same, i.e., 18-15/13-7:1:0 (inh15_SSP5) and 18-17/13-7:1:0 (inh15_SSP6). We did not find a significant difference in Ct values for 10^5^ copies of mutant, or 10^5^ copies of WT obtained with primers inh15_SSP4 and inh15_SSP5, which have bridge lengths of 14 and 15 bp, respectively. Primer inh15_SSP6 with a bridge length of 17 bp formed a bigger and more asymmetric bubble, delaying the amplification of 10^5^ copies of mutant or 10^5^ copies of WT by 1 cycle. The window of discrimination (ΔCt) between the Ct for the mutant and the WT was similar for these two primers.

Furthermore, we tested different bridge-intervening sequence length combinations—20/8, 8/8, and 8/20 (inh15_SSP7-9)—in the SuperSelective primer designs. SuperSelective primers with a long bridge but short intervening sequence 18-20/8-7:1:0 (SSP7), short bridge–short intervening sequence 18-8/8-7:1:0 (SSP8), and short bridge–long intervening sequence 18-8/20-7:1:0 (SSP9) were tested at Tm 60–66 °C, while all other RT-PCR conditions were maintained similar to the above (Table S1). Amplification of mutant target using SuperSelective primer with a symmetric bubble 18-8/8-7:1:0 (SSP8) was the least affected by changes in Tm, although the window of discrimination (ΔCt) between the Ct for the mutant and the Ct for the WT increased with increasing temperature (Figure S2a). Amplification of the mutant target using both SuperSelective primers forming an asymmetric bubble (18-20/8-7:1:0 and 18-8/20-7:1:0) was delayed according to the Tm of the assay. Only 18-20/8-7:1:0 suppressed the amplification of the WT pDNA template (at Tm 66 °C) but also caused a delay (5 cycles) in the amplification of the intended mutant target (Figure S2b).

### 2.4. Changes in Foot Design

In some of the potential SuperSelective primer designs tested previously, we introduced a mismatch in the foot sequence at the fourth position from the interrogating nucleotide, i.e., 18-14/14-6:1:0 (inh15_SSP14), 18-14/13-7:1:0 (inh15_SSP15), and 18-20/8-7:1:0 (inh15_SSP14- inh15_SSP16) to destabilize the binding of the foot sequence with the WT DNA. We also tested SuperSelective primers 18-14/14-3:2:1, 18-14/14-6:2:1, and 18-14/14-6:2:0 (inh15_SSP23 to inh15_25, respectively) by introducing a mismatch nucleotide immediately after the interrogating nucleotide. In theory, this design would weaken the binding of the foot sequence with its target, with a more pronounced effect on the WT target with two mismatches compared to the mutant with a single mismatch. We also investigated the effect of varying the location of the interrogating nucleotide in the foot sequence on the primer’s ability to discriminate mutant templates from WT templates using primer designs 18-14/14-6:1:2, 18-14/13-7:1:2, 18-20/8-7:1:2, and 18-14/14-3:2:1 (inh15_SSP19 to inh15_SSP22, respectively). Results from the PCR assays indicate that the variations in foot sequence design aimed to prevent the amplification of WT DNA led to a 8–16 cycle delay in the amplification of the mutant target.

### 2.5. SuperSelective Reverse Primers

To decrease the binding of SuperSelective forward primers paired with conventional reverse primers to WT DNA, the SuperSelective forward primers were paired with SuperSelective reverse primers [31]. We designed SuperSelective reverse primers 18-14/14-6:1:0 (inh15_SSP10) and 18-14/13-7:1:0 (inh15_SSP11) with varying foot length (7/8 nt), 18-14/8-6:1:0 (inh15_SSP12) and 18-14/8-7:1:0 (inh15_SSP13) with varying foot length (7/8 nt) plus a shorter intervening sequence (8 bp), and 20-14/14-6:1:0 (inh15_SSP17) and Rev 20-14/13-7:1:0 (inh15_SSP18) with varying foot length (7/8 nt) and a longer anchor (20 nt). These primers were paired with SuperSelective primers 18-14/13-7:1:0 (inh15_SSP4) and 18-20/8-7:1:0 (inh15_SSP7) or their respective anchor sequences, which acted as conventional forward primers. The effect of foot length and intervening sequence on a reverse SuperSelective primer paired with a conventional forward primer was as shown above for a forward SuperSelective primer paired with a conventional reverse primer. We compared the Ct values for detecting 10^5^ copies of mutant and 10^5^ copies of WT with each of these SuperSelective primers paired either with a conventional second primer to their pairing with another SuperSelective primer. Results show that using two SuperSelective primers drastically increased the window of discrimination (ΔCt) for mutant and WT targets from <5 to 8–13, yet it did not completely suppress the amplification of WT templates.

Some of these variations in SuperSelective primer design were also tested on other mutations in the panel, i.e., *inhA* (-8T→A, -17G→T), *katG* (S315T, AGC→ACA, and AGC→ACC), and *rpoB* (D516V, GAC→GTC; H526D, CAC→GAC; H526Y, CAC→TAC; and S531L, TCG→TTG) (Table S2). All the tested SuperSelective primer designs and combinations amplified the unintended WT target or the suppression of WT DNA was accompanied by a delay in amplification of 10^5^ copies of mutant and/or suppression of amplification of ten and one copy of mutant. We repeatedly observed an overlap of low copy number mutant (ten and one) and WT amplification peaks. It should be noted that the Sybr Green reagent used to monitor amplification in these RT-PCR assays is a DNA-intercalating dye that detects specific and non-specific amplification products. Therefore, to confirm the specificity of the amplified products tested by Sybr Green, a subset of SuperSelective primers were tested for amplification using molecular beacon probes.

### 2.6. Validation of RT-PCR Assays for Mutant Detection with Molecular Beacon Probes

Amplification of WT DNA can lead to a false positive sample detection in RT-PCR assays, particularly for clinical samples with low copies of mutant DNA and high copies of WT DNA. To confirm whether the amplification products (from WT DNA) in our SuperSelective PCR assays were the intended amplification products and not the amplification of non-specific products, such as primer-dimers, we designed and tested amplification-product-specific molecular beacon probes in the PCR assays [32]. Since non-hybridized molecular beacons are virtually non-fluorescent and hybridized molecular beacons fluoresce brightly in their characteristic color [32,33], the amount of amplicon present during the annealing phase of each amplification cycle is automatically measured by the spectrofluorometric thermal cycler in which the PCR assays are carried out.

### 2.7. Targeting Closely Localized Mutations

Of the panel of mutations selected for this study, both *katG* mutations AGC→ACA and AGC→ACC are present at the same codon (315). The anchor and foot sequences of the SuperSelective primers designed to detect these mutations overlapped with each other. Similarly, *inhA* mutations -8T→A, -15C→T, and -17G→T are co-localized in the promoter region and *rpoB* mutations D516V (GAC→GTC), H526D (CAC→GAC), H526Y (CAC→TAC), and S531L (TCG→TTG) in the RIF-resistance-determining region (RRDR) with overlapping regions of the primer. The amplification products of both *katG* mutations, three *inhA* promoter mutations, and four *rpoB* mutations also had overlapping nucleotide sequences. These regions were exploited to design the molecular beacon probes to detect mutations. Thus, we could detect both *katG* mutations using the same/single probe. Similarly, we designed one probe for the identification of three mutations in the *inhA* promoter region, i.e., -8T→A, -15C→T, -17G→T, and one probe for all the *rpoB* mutations D516V (GAC→GTC), H526D (CAC→GAC), H526Y (CAC→TAC), and S531L (TCG→TTG). Thus, three molecular beacon probes were utilized to detect nine mutations. We designed the following probes, which included a unique sequence at both ends of the probe (in italics), to form the stem region and labeled the probe with FAM at the 5′ end and a quencher BHQ-1 at the 3′ end.

*katG*: 5′ FAM-CGCTCG GACGAACACCCCGACGAAAT CGAGCG-BHQ-1 3′

*inhA*: 5′ FAM-CGCTCG CCGGGCCGAAATCGGTATGT CGAGCG-BHQ-1 3′

*rpoB*: 5′ FAM-CGCTCG GAATTGGCTCAGCTGGCTGG CGAGCG-BHQ-1 3′

### 2.8. Use of Asymmetric PCR Conditions

We conducted three separate RT-PCR assays to optimize the molecular beacon probe concentrations for *katG*, *inhA*, and *rpoB*. The concentration of each probe was optimized for at least one mutation from the group for which it was specific. Thus, the molecular beacon probe *katG* was optimized for the detection of *katG* S315T (AGC→ACA), the *inhA* probe for *inhA* promoter mutation -8T→A, and the *rpoB* probe for *rpoB* mutation D516V (GAC→GTC). RT-PCR assays were carried out using 10^5^ copies of linearized mutant plasmid in three sets of reactions with variable concentrations of the probe, i.e., 0.1, 0.125, and 0.25 µM. We used 0.1 µM each of forward and reverse primers in these assays. Fluorescence intensity with the 0.25 µM probe was the highest as expected (>2-fold), while there was no significant difference between relative fluorescence units (RFU) obtained with 0.1 and 0.125 µM probes. Figure S3 demonstrates an example of the increase in fluorescence signal intensity with increasing concentration of *inhA* probe with 10^5^ copies of *inhA* -8T→A mutants, using SuperSelective primer 18-14/14-6:1:0.

In order to avoid probe crosstalk in multiplex reactions with a very high amount of probe, we tested asymmetric primer concentrations to enhance fluorescence signal intensity in the reactions to control the amount of molecular beacon probe. RT-PCR assays were performed in two sets with SuperSelective primer and conventional reverse primer in the ratios 1:5 and 1:10 on 10^5^ copies of mutant target DNA using a 0.1 µM probe. SuperSelective primer and conventional reverse primer in the ratio 1:1 (symmetric PCR, 0.1 µM each of forward and reverse primer) were used as a control for these reactions. We observed a 2.5-3-fold rise in fluorescence signal intensity under both tested asymmetric PCR conditions, although there was no significant difference between primer ratios 1:5 and 1:10. Figure S4 shows the amplification of *rpoB* D516V GAC→GTC with SuperSelective primer 18-14/13-7:1:0 paired with a conventional reverse primer in 1:1, 1:5, and 1:10 ratios on 10^5^ copies of mutant target DNA. These experiments demonstrate the advantage of using non-symmetric primer concentrations in RT-PCR assays over symmetric primer concentrations.

We extended our observations from the above experiments to other mutations included in this study, *katG* S315T AGC→ACC, *inhA* promoter -15C→T and -17G→T, *rpoB* H526D (CAC→GAC), H526Y (CAC→TAC), and S531L (TCG→TTG). All the subsequent monoplex RT-PCR assays with molecular beacon probes were performed under asymmetric PCR conditions utilizing 0.1 µM and 0.5 µM of forward and reverse primer, respectively, and 0.1 µM of the probe to maximize the fluorescence signal intensity. We further optimized the individual probe concentrations for the multiplexed assays (discussed later).

### 2.9. Selective Amplification of the Mutant from Mixed DNA

To detect each of the nine mutations in our selected panel of target mutant genes and to determine their selectivity, one to five SuperSelective primers were tested. RT-PCR experiments were performed using linearized mutant and WT pDNA templates in the ratios 10^5^-1:10^5^ (Mutant:WT) and 10^5^-1:10^4^ (Mutant:WT). Each primer was tested at least twice on duplicate samples. Low copy number mutants (ten and one) and WT samples were tested on three to eight replicates.

RT-PCR assay with the molecular beacon probes confirmed that the amplification products initiated from WT DNA templates (with 10^5^ copies of WT DNA) resulted from specific amplification. Under the tested assay conditions, SuperSelective primers (Table 1) successfully suppressed the amplification of 10^4^ copies of WT DNA while amplifying the mutant to one copy. Primers were tested for up to 60 RT-PCR cycles to check for the appearance of late amplification signals (after Ct 50) from WT. Using the selected SuperSelective primers (Table 1), we could detect all nine mutations in our panel with absolute specificity. We could detect down to one copy of mutant within 50 cycles of the RT-PCR, with detection of WT (up to 10^4^ copies) suppressed until 60 cycles. These observations confirmed that SuperSelective primers are highly specific for amplifying the target mutant DNA (specificity 100%) while suppressing the amplification of up to 10^4^ copies of WT DNA.

### 2.10. Highly Sensitive Method of Mutant Detection in a Background of Susceptible M. tuberculosis DNA

The parameters of SuperSelective primer design and RT-PCR assay conditions were optimized initially using linearized plasmids containing the mutation of interest. Plasmids, which are limited in their total length, generate a low probability of SuperSelective primers to find non-specific binding sites, which will increase many-fold in a sample containing the genome of *M. tuberculosis*. To validate our SuperSelective primer designs and RT-PCR assay conditions, we tested selected SuperSelective forward primer and conventional reverse primer combinations (Table 1) on several *M. tuberculosis* gDNA samples containing the mutations of interest. We tested these conditions on 15 mutant gDNA samples containing the panel of mutations, as shown in Table 1. Of these, ten gDNA samples were MDR with *katG* S315T/*inhA*-15/*inhA*-17 mutation and one of the four RRDR mutations. (Table 1).

**Figure 1:**
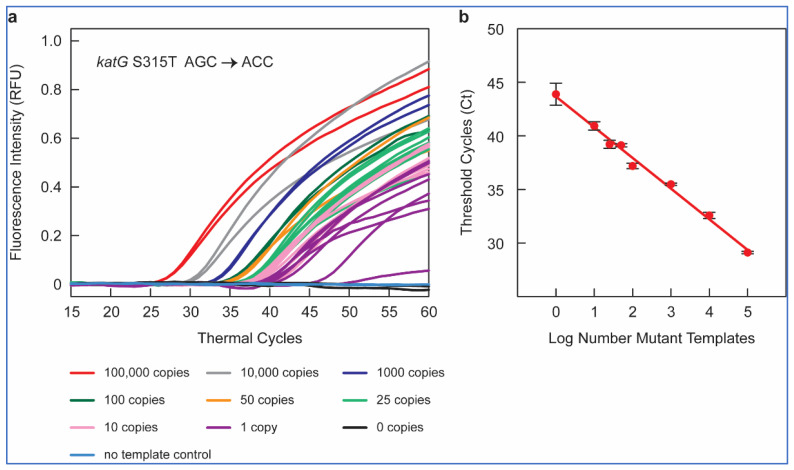
SuperSelective primer-based detection of *katG* S315T AGC > ACC mutant sequences in the presence of abundant WT sequences. (**a**) Results of RT-PCR assays to detect *katG* S315T AGC > ACC mutations with a FAM-labeled molecular beacon probe utilizing the SuperSelective primer 20-14/14-5:1:0 with gDNA as a template. The figure shows amplification of 10^5^ (red), 10^4^ (grey), 10^3^ (blue), 10^2^ (dark green), 50 (orange), 25 (light green), 10 (pink), 1 (violet) copies of mutant DNA template in a background of 10^4^ copies of WT DNA template; 10^4^ copies of WT (black) and nuclease-free water (yellow) were used as control. (**b**) The threshold cycle measured for each reaction that contained mutant templates is plotted as a function of the logarithm of the number of mutant templates initially present in each reaction.

We tested each of the selected SuperSelective primer and RT-PCR assay conditions on 10^5^ to one copy of mutant gDNA in a background of 10^4^ copies of WT *M. tuberculosis* H37Rv gDNA. We tested two replicates for high copy numbers (10^5^, 10^4^, 10^3^, 10^2^, 50) of mutant, eight replicates for the low copy numbers (25, 10, 1) of mutant, and at least four replicates of 10^4^ copies of WT. Consistent with our findings in the plasmid-based assays (Table 1), we were able to amplify up to one copy of mutant DNA (in at least two out of eight replicates) for all the nine mutations in *katG*, *inhA*, and *rpoB*. Ten copies of mutant DNA were amplified in at least four out of eight replicates and twenty-five copies in at least six out of eight replicates, while eight out of eight replicates were positively detected for fifty copies of mutant DNA or more. Figure 1a shows the amplification peaks obtained in RT-PCR assays containing 10^5^, 10^4^, 10^3^, 10^2^, fifty, twenty-five, ten, one, and zero copies of *katG* S315T AGC > ACC mutant gDNA sample in a background of 10^4^ copies of WT *M. tuberculosis* H37Rv DNA, utilizing the SuperSelective primer 20-14/14-5:1:0 combined with a conventional reverse primer and using a FAM-labeled molecular beacon probe for the detection.

Thus, our SuperSelective primer-based RT-PCR assays could detect one in ten thousand copies of mutant DNA for all nine gene mutations included in our panel, confirming the ultra-high sensitivity (0.01%) of these SuperSelective primers for the detection of INH and RIF resistance in *M. tuberculosis*.

### 2.11. Quantifiable Mutant Detection

The clinical goal of these RT-PCR assays that utilize SuperSelective primers is to measure the relative abundance of mutant DNA fragments relevant to INH and RIF resistance in TB in the context of the amount of WT *M. tuberculosis* DNA present in the sample. In our RT-PCR assays, control reactions that contained no template DNA did not produce any false amplicons, such as primer-dimers, despite the longer length of the SuperSelective primers. The reaction containing 10^4^ WT templates and no mutant templates was suppressed to such an extent that it did not produce a significant number of amplicons until after 50 cycles of amplification had been carried out (Figure 1a).

To examine the potential of SuperSelective primer pairs for mutant quantification, we compared the mean Ct values (in an RT-PCR assay) with each SuperSelective primer to the mutant copy number tested. The mean Ct values were inversely linearly proportional to the logarithm of the number of copies of mutant target DNA (Figure 1b). This inverse linear relationship between the logarithm of the number of mutant targets initially present in a sample and the Ct value observed for that sample is the hallmark of quantitative exponential amplification assays. Thus, RT-PCR assays employing SuperSelective primer pairs could be utilized to determine the actual amount of mutant target DNA even in a high background (10^4^ copies) of WT DNA, based only on Ct values. These results demonstrate the potential of SuperSelective primer in RT-PCR assays to not only detect but also quantify the amount of rare mutant DNA in the presence of abundant WT *M. tuberculosis* DNA, providing a novel and powerful tool for detecting and quantifying heteroresistance in TB patients.

### 2.12. Multiplexed Assay

The mutations causing INH and RIF resistance in *M. tuberculosis* are localized within a compact region of katG gene, *inhA* promoter, and RRDR of the rpoB gene. For mutant sequences occurring in separate genes, multiplex real-time PCR assays can be designed relatively easily to quantitate the amount of each mutant gene in the same sample. SuperSelective primers for these mutations would have different anchor and foot sequences. The amplicons generated from each mutant gene will possess a unique sequence that can be easily distinguished from the sequences of amplicons generated from the other mutant genes, making it easy to distinguish these amplicons from each other through the use of differently colored molecular beacon probes. Thus, we decided to multiplex the RT-PCR assays to detect mutations present on different drug resistance genes.

Amongst the drug-resistant TB cases, *katG* S315T mutation is the most common mutation and *inhA* mutation (-15C→T) is the second most frequently observed mutation associated with INH resistance [34], while *rpoB* mutation S531L (TCG→TTG) is the most widespread mutation responsible for high-level resistance to RIF [35]. Considering the importance of these three mutations in MDR-TB cases, we designed a prototype multiplex assay incorporating SuperSelective primers to simultaneously detect katG gene S315T AGC→ACA, *inhA* -15 C→T, and S531L TCG→TTG.

### 2.13. Optimization of Assay Components

For the simultaneous detection of katG gene S315T AGC→ACA, *inhA* -15 C→T, and S531L TCG→TTG, we utilized SuperSelective primers having LoD of one copy of mutant in the corresponding monoplex assays (Table 1) and tested the efficiency and specificity of these primers in a multiplexed environment. To ensure that the selected primer pairs do not have any cross-reactivity with each other, we tested these three primer pairs in various possible combinations. For example, we tested the *katG* S315T AGC→ACA and *inhA* -15 C→T primer pair in a duplex RT-PCR assay on 10^5^ copies of *katG* S315T AGC→ACA mutation containing linearized pDNA to study the effect of *inhA* -15 C→T primer pair addition on the amplification threshold of *katG* S315T AGC→ACA mutant. A monoplex RT-PCR assay with a *katG* S315T AGC→ACA primer pair and 10^5^ copies of *katG* S315T mutation containing linearized pDNA was used as a control. Sybr Green was used to monitor the amplification of these reactions. We also studied the effect of *katG* S315T AGC→ACA primer pair addition on the amplification threshold of *inhA* -15 C→T by using *inhA* -15 C→T mutation containing linearized pDNA.

Similarly, *katG*-*rpoB*, *inhA*-*rpoB*, and *katG*-*inhA*-*rpoB* primer pairs were also tested. The *rpoB* primer pair addition delayed the amplification threshold of both *katG* S315T AGC→ACA and *inhA* -15 C→T mutants in the duplex assays, but the Ct values in the three-plex assays were unaffected. Thus, these three primer pairs were utilized to further optimize the multiplex RT-PCR assay. For these three-plex RT-PCR assays, we used three molecular beacon probes specific for amplicons generated by SuperSelective primers corresponding to *katG*, *inhA*, and *rpoB*. Each probe was labeled with a different fluorophore to detect signals for each mutation in a different optical channel.

*katG*: 5′ FAM-CGCTCG GACGAACACCCCGACGAAAT CGAGCG-BHQ-1 3′

*inhA*: 5′ Cal Fluor Red 610-CGCTCG CCGGGCCGAAATCGGTATGT CGAGCG-BHQ-2 3′

*rpoB*: 5′ Quasar 670-CGCTCG GAATTGGCTCAGCTGGCTGG CGAGCG-BHQ-2 3′

Probe concentrations were re-optimized for multiplex RT-PCR assays. Asymmetric RT-PCR assays were carried out on a Bio-Rad CFX96 Real-Time System in a final volume of 25 µL with 1X PCR buffer supplemented with 25 mM TMAC and 0.25% Tween20, 3 mM MgCl2, 0.05 U/µL Platinum Taq DNA polymerase, 0.25 mM dNTPs, each forward primer (*katG*, *inhA*, *rpoB*) 0.1 µM, each reverse primer (*katG*, *inhA*, *rpoB*) 0.5 µM, probe *katG* 0.25 µM, probe *inhA* 0.1 µM, and probe *rpoB* 0.25 µM. Amplification was performed for 60 cycles with Tm 60 °C for 20 s and extension at 72 °C for 20 s.

### 2.14. Selective Amplification

To ensure that each amplicon is only copied by its “correct” SuperSelective primer under the three-plex assay conditions, we carried out three sets of experiments, each with linearized plasmids containing *katG* S315T AGC→ACA, *inhA* -15 C→T, or S531L TCG→TTG mutation. We tested 10^5^-1:10^4^ copies of mutant:WT pDNA. Each condition was tested on at least two replicates.

**Figure 2:**
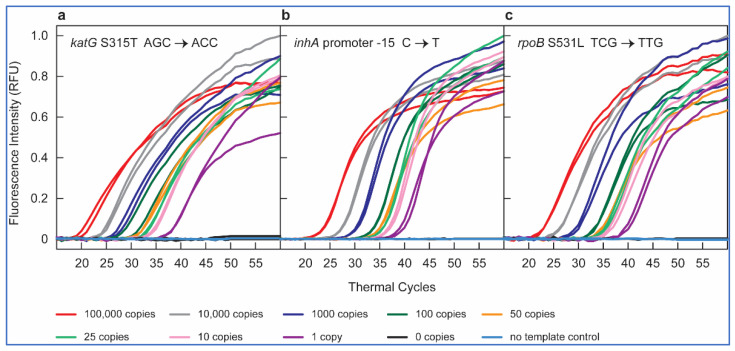
Results of multiplex RT-PCR assays to detect *katG* S315T AGC > ACC, *inhA* -15 C→T, and S531L TCG→TTG mutations utilizing the SuperSelective primers 20-14/14-5:1:0, 18-14/13-7:1:0, and 20-14/13-7:1:0, respectively, with pDNA used as a template. Molecular beacon probes labeled with FAM, CFR610, and Q670 were used to detect amplification signals. The figure illustrates amplification of 10^5^ (red), 10^4^ (grey), 10^3^ (blue), 10^2^ (dark green), 50 (orange), 25 (light green), 10 (pink), 1 (violet) copies of mutant DNA template in a background of 10^4^ copies of WT DNA template; 10^4^ copies of WT (black) and nuclease-free water (yellow) were used as control. The figure shows amplification peaks of (**a**) *katG* S315T AGC > ACC pDNA detected in FAM channel, (**b**) *inhA* -15 C→T pDNA detected in CFR610 channel, and (**c**) S531L TCG→TTG detected in Q670 channel. There was no amplification in non-specific channels; hence, the data has been excluded from these graphs.

In our first set of RT-PCR multiplex assays, with *katG* S315T AGC→ACA mutant-containing pDNA, we detected amplification signals only in the FAM channel (Figure 2a) down to one copy of mutant DNA in a background of 10^4^ copies of WT DNA. No signals were detected in CAL Fluor Red 610 (CFR610) and Quasar 670 (Q670) channels. Similarly, in multiplex reactions with pDNA containing *inhA* -15 C→T and S531L TCG→TTG mutant, fluorescence was recorded only in the CFR610 (Figure 2b) and Q670 (Figure 2c) channels, respectively, while no amplification signal was observed in the non-specific channels. These results confirmed that the SuperSelective primers detect only specific mutants even in the presence of abundant WT DNA fragments. None of the primer pairs cross-reacted to amplify a non-specific target, and they completely suppressed the unintended WT target in the background, signifying a high specificity of SuperSelective primers even in a multiplexed environment.

### 2.15. Sensitivity on Clinical gDNA with Potential Sputum Carryover

To expand the utility of our optimized SuperSelective PCR to clinical applications, we tested 23 *M. tuberculosis* gDNA samples isolated from the sputum of pulmonary TB patients. The sputum samples were obtained from microbiologically, radiologically, and clinically confirmed TB cases (Table 2). The samples were single-blinded to the status of phenotypic or genotypic drug resistance in these *M. tuberculosis* gDNA samples.

We first assessed the total amount of DNA present in each of these 23 clinical gDNA samples by amplifying a reference *M. tuberculosis* WT sequence, 16S rRNA (rrs), present in the sample and unrelated to the mutations of interest. The generation of amplicons from this reference sequence served as a quality indicator control for the gDNA samples. The RT-PCR assays were performed using the conventional forward and reverse primer to amplify a region of rrs gene using Sybr Green to monitor amplification. The Ct value of these WT amplicons reflects the amount of DNA present in the sample. The number of copies of rrs was calculated using the slope and intercept of the standard curve, which was plotted using 10^5^-1 copies of WT H37Rv gDNA. If the number of genomes turned out to be zero or lower than a predetermined value, the assay results would have been ignored due to there being too little DNA in the sample for the rare target mutations, if they exist, to be present.

This assay confirmed that all the clinical samples had detectable copies of the rrs, ranging from 33 to 25,000. Next, we performed RT-PCR using the mutation-specific SuperSelective primers (Table 1) to determine the number of mutant DNA copies present in each of these 23 clinical *M. tuberculosis* gDNA samples, with mutant gDNA used to prepare the standards at copy numbers ranging from 1 to 10^5^. We first plotted the standard curve using mean Ct values versus the logarithm of the number of copies of mutant target DNA for each mutation (as shown in Figure 1b). Next, the clinical *M. tuberculosis* gDNA samples were subjected to RT-PCR with the SuperSelective primers, and the number of copies of each mutant gene was calculated based on the slope and intercept of the standard curve.

To validate our assessment of drug resistance detection based on SuperSelective primers, sections of katG, inhA, and rpoB genes containing the mutations of interest were sequenced in 11/23 samples (Table 2). Eight out of twenty-three samples were also tested for phenotypic drug susceptibility using pDST. Based on the RT-PCR assay using SuperSelective primers, six out of twenty-three samples were found to be WT, two of which were also confirmed by pDST. Using SuperSelective primers, six out of twenty-three samples were found to be INH-monoresistant, including five with *katG* S315T AGC→ACA mutation and one with *inhA* -15 C→T mutation. Of these, one of the *katG* mutant sample was confirmed to be INH-resistant by pDST. Using SuperSelective primers, we also identified 11/23 clinical gDNA samples as MDR with mutations conferring both INH and RIF resistance. Five of eleven of these samples had a complete agreement between the SuperSelective primer RT-PCR assay and sequencing, and three out of eleven tested by pDST were confirmed to be INH- and RIF-resistant (Table 2). In three of the eleven samples (AVR2, AVR11, and AVS35), there was a discordance between the SuperSelective primer RT-PCR assay and sequencing. The SuperSelective primer RT-PCR assay detected sample AVR2 as MDR, demonstrating five copies of *katG* S315T AGC→ACA and thirty-eight copies of *inhA* -15 C→T mutant DNA in a total sample of two hundred fifty-four gDNA copies. Neither of these INH mutations were detected by DNA sequencing; thus, this sample would be considered as RIF-monoresistant, rather than MDR. With the SuperSelective primer RT-PCR assay, sample AVR11 had two and four copies of *katG* S315T AGC→ACA and *rpoB* S531L TCG→TTG mutant DNA, respectively, in a total background of over twenty-four thousand copies of gDNA. This sample was detected as WT by sequencing, yet pDST results of this sample classified it as MDR. In another sample (AVS35), pDST and sequencing failed to detect drug resistance. However, SuperSelective primers identified ten and two copies of *inhA* -15 C→T and *rpoB* S531L TCG→TTG mutant DNA, respectively, with rrs copies being >5500 in this sample.

While targeted sequencing is known to detect heteroresistance with a limit of detection (LoD) of 10%, SuperSelective primers were able to detect specific mutants as low as two copies in the presence of abundant DNA fragments from the entire *M. tuberculosis* genome with exceptional sensitivity, due to the suppression of amplification of the abundant WT DNA fragments.

## 3. Discussion

We report the successful use of SuperSelective PCR primers for detecting RIF and INH heteroresistance in TB with a detection limit of 0.01% for the mutant DNA/WT DNA ratio. Our method shows a 10-fold improvement in detecting mutant gene copies in the presence of abundant WT genes of *M. tuberculosis* genome over the most sensitive methods reported to date, such as digital PCR [10].

We present nine SuperSelective primer designs which, under optimized RT-PCR assay conditions, can detect nine mutations commonly associated with RIF and INH resistance in TB disease, including *katG* (S315T, AGC→ACA, and AGC→ACC), *inhA* promoter (-8T→A, -15C→T, and -17G→T), and *rpoB* (D516V, GAC→GTC; H526D, CAC→GAC; H526Y, CAC→TAC; and S531L, TCG→TTG). After generating primers for mutations associated with resistance to INH and RIF, we performed clinical validation of the approach in *M. tuberculosis* DNA isolated from sputum samples obtained from TB patients, which included a mixture of drug-sensitive and drug-resistant cases. While our SuperSelective primer-based approach confirmed the presence of mutant DNA in patients with sequencing results demonstrating the mutation of interest in all instances, we also detected low copy numbers of mutant DNA among several patients with putative WT DNA by sequencing, confirming the presence of heteroresistant *M. tuberculosis* below the threshold that can be amplified by standard methods of DNA sequencing.

Heteroresistance, known as the detection of both the mutant and the wild-type strains, is considered to be the early stage in the development of drug-resistant TB and is reflective of the slow evolution of bacteria from a sensitive to resistant profile [36]. Heteroresistance was initially attributed to infections with multiple infecting *M. tuberculosis* strains (with mixed phenotypic resistance patterns); strains from different lung regions demonstrate different drug susceptibilities. In recent years, the implementation of molecular diagnostic techniques has led to an alternate conceptualization of heteroresistance, recognizing that infection by a single *M. tuberculosis* isolate may contain both mutant (drug-resistant) and wild-type (drug-susceptible) genomic DNA of the pathogen. Standard phenotypic culture-based drug resistance assays lead to the loss of these heteroresistant variants during the subculture process, interfering with our understanding of this phenomenon. The sensitivity of GenoType MTBDRplus and LPA Nipro for heteroresistance detection is about 5% [12,14]. While with more advanced techniques, such as digital PCR, the detection threshold can reach up to 0.1% mutant:WT [10], these assays require sophisticated equipment and/or are more expensive to implement in TB-endemic countries. In contrast, our method involves a carefully designed PCR primer strategy that can be used in a standard RT-PCR machine. However, as with all other primer- and probe-based amplification assays, and unlike sequencing-based methods, it is necessary to know the exact nature of the polymorphism to develop a SuperSelective primer-based RT-PCR assay.

Early identification of heteroresistant TB may support efforts to optimize TB therapy on an individual basis, for example, with adherence interventions or clinical decisions on drug selection, dosing, or therapeutic drug monitoring. SuperSelective primers, despite their extraordinary discriminatory ability (higher than conventional methods), are supremely easy to use. The only difference between these assays and conventional multiplex RT-PCR assays is the substitution of SuperSelective primers for conventional primers, while the sample preparation, amplification, and assessment are carried out in the same manner. Thus, RT-PCR assays with SuperSelective primers can be used as an accessory technique for resistance detection along with other methods of TB detection. Although the current study was performed using sputum samples from primary TB cases, utility of SuperSelective PCR primers can be extended to re-treatment cases, which usually have a higher probability of a low to moderate number of copies of mutant DNA in the sputum samples and are relatively easier to detect.

In summary, we report the successful development and application of “SuperSelective” primer-based RT-PCR to detect gene mutations in *M. tuberculosis* associated with heteroresistance in an abundant wild-type background. Improved diagnostic tools, such as the one described here, would enable better and improved therapeutic management of drug-sensitive and drug-resistant TB cases in general and heteroresistant TB cases in particular.

## 4. Materials and Methods

### 4.1. Selected Antibiotic-Drug-Resistant Mutations

SuperSelective primers were designed to amplify mutant *M. tuberculosis* DNA corresponding to INH and RIF resistance in a WT background. For INH resistance, SuperSelective primers were designed to target *katG* (S315T, AGC→ACA, and AGC→ACC) and the promoter region of *inhA* (-8T→A, -15C→T, and -17G→T) [25,26,27]. For RIF resistance, the primers were designed to focus on the most common mutations in the RRDR of *rpoB* (D516V, GAC→GTC; H526D, CAC→GAC; H526Y, CAC→TAC; and S531L, TCG→TTG) [37].

### 4.2. Structure and Functionality of SuperSelective Primers

The SuperSelective PCR primers were designed to have a minimal probability of initiating synthesis on WT sequences, even when the only difference between the mutant and the WT targets was a single-nucleotide polymorphism. The SuperSelective primer designs were adapted from our previous reports [25,38]. Briefly, SuperSelective primers used in the present study consist of the 3 sequence segments in order from 5′ to 3′: (i) an anchor sequence complementary to the target DNA that is sufficiently long to ensure strong hybridization to its DNA target fragment under PCR annealing conditions; (ii) a distinctive bridge sequence, which is non-complementary to the corresponding intervening sequence in the DNA target fragment; and (iii) a short 3′-foot sequence that is entirely complementary to the corresponding sequence in the mutant DNA target fragments, but is discrepant to the corresponding sequence in the related WT DNA fragments.

**Figure 3:**
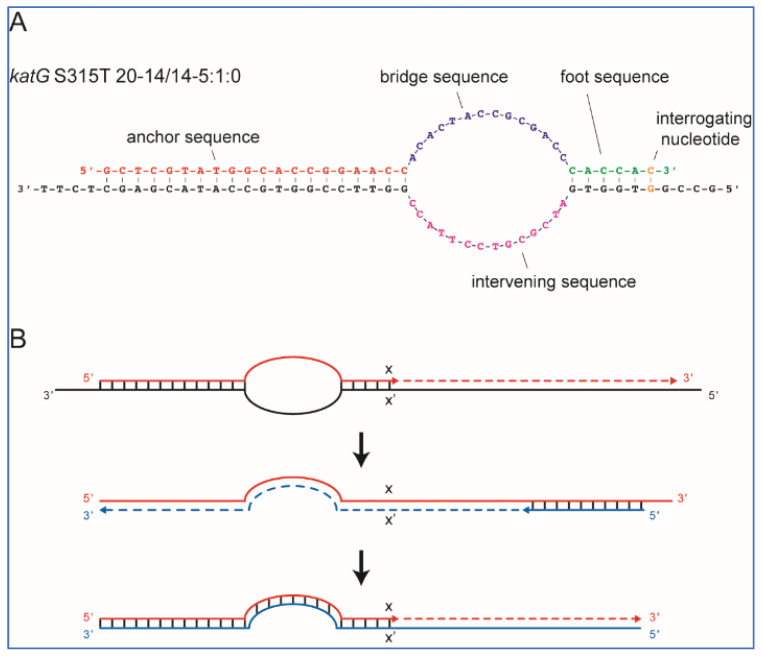
Structure and principle of operation of SuperSelective primers. (**A**) Structure of a SuperSelective primer for detecting *katG* S315T (AGC→ACA) mutant sequences in the presence of *katG* WT sequences. (**B**) Principle of operation of SuperSelective primers. The selective step occurs only when a SuperSelective primer hybridizes to a DNA (−) template fragment present in the sample. Due to the small size of the foot sequence, the probability of initiation of a (+) amplicon is significantly greater if the target sequence of the foot in the (−) template fragment is a completely complementary mutant (x) sequence than if the target sequence of the foot in the (−) template fragment is a mismatched WT sequence. Suppose (+) amplicon synthesis does occur. In that case, the resulting (+) amplicon serves as a template for a conventional reverse primer and is efficiently copied during the next thermal cycle, generating a (−) amplicon in which the complement of the unique bridge sequence that was present in the SuperSelective primer is substituted for the intervening sequence that was present in the original (−) template fragment. As a result, in subsequent thermal cycles, the entire SuperSelective primer sequence is complementary to the (−) amplicon strands, and exponential amplification occurs efficiently and can be followed in real time.

In a PCR assay, the function of a SuperSelective primer is to maintain a delicate balance between the anchor sequence (long 5′ segment), which promotes its efficient binding to a gene of interest, and the foot sequence (short 3′ segment), which restricts binding to the subsequence that includes the target mutation. Complementarity between the “interrogating nucleotide” in the foot to the corresponding nucleotide in the mutant target sequence makes the foot and mutant target perfectly compatible and leads to priming of the synthesis of an amplicon, while the mismatch at the corresponding nucleotide in the WT target sequence inhibits the amplification of the WT target. DNA-DNA hybrids formed by the anchor sequence with target DNA and the short foot sequence with target DNA are separated from each other by a single-stranded bubble formed by the non-complimentary bridge sequence (which should not form any secondary structures) and the intervening sequence in the DNA target fragment. This bubble effectively separates the primer’s polymerization initiation from the primer’s target recognition. Figure 3A shows an example of a SuperSelective primer bound to its mutant target sequence. This particular primer was designed to selectively amplify DNA fragments containing *katG* S315T (AGC→ACA) single-nucleotide polymorphism, which is commonly associated with INH resistance in TB patients.

The nomenclature for primer labeling was as we described earlier [25]. For example, a SuperSelective primer 20-14/14-5:1:0 (shown in Figure 3A) indicates that the anchor sequence is 20 nucleotides long, the bridge sequence is 14 nucleotides long (across from an intervening sequence in the template that is 14 nucleotides long), and the foot sequence is 6 nucleotides long, with one interrogating nucleotide located at the last position from the primer’s 3′ end.

In an RT-PCR assay with a SuperSelective primer, the selective step occurs when a SuperSelective primer (designed against DNA (−) strand template) is bound to the original DNA sample being analyzed. The foot sequence of a SuperSelective primer initiates the synthesis of an amplicon, and as the reaction proceeds, the entire sequence of the SuperSelective primer (including the “artificial” bridge sequence) is incorporated into that (+) amplicon. In the successive thermal cycles, the resulting amplicons are amplified efficiently in the normal manner, with the entire SuperSelective primer sequence serving as a long conventional primer that is completely complementary to the (−) amplicon, which includes the complement of the primer’s bridge sequence in place of the intervening sequence that was present in the original template (Figure 3B).

### 4.3. Primers, Molecular Beacons, and PCR Reagents

In all assays, a SuperSelective forward primer was paired with a conventional reverse primer unless mentioned otherwise. SuperSelective primer sequences were assessed using the Mfold web server [39] and the OligoAnalyzer computer program (Integrated DNA Technologies, Coralville, IA, USA) to ensure these sequences are unlikely to form internal secondary structures like hairpin loops, self-dimers, or heterodimers with conventional reverse primers. Conventional and SuperSelective primers were purchased from Integrated DNA Technologies. Molecular beacon probes were purchased from LGC Biosearch Technologies (Petaluma, CA, USA). Platinum Taq DNA Polymerase, deoxynucleotide triphosphates (dNTPs), and Sybr Green were purchased from Thermo Fisher Scientific, Inc. (Waltham, MA, USA). Nuclease-free water was purchased from Ambion, Inc. (Austin, TX, USA).

### 4.4. Target DNA

Complete WT and mutant (AGC→ACA) katG genes of *M. tuberculosis* were cloned into the vector pcDNA3.1 [40] and used as the source of pDNA for assays. For the optimization of assays to detect AGC→ACC mutation, plasmids (pUC) containing a 584 bp subsequence of katG gene (WT and AGC→ACC) were synthesized (Integrated DNA Technologies). To detect *inhA* promoter mutations, plasmids (pUC) containing a 387 bp region covering mutations (-8T→A, -15C→T, and -17G→T) and the corresponding WT were synthesized (Integrated DNA Technologies). To detect *rpoB* mutations, a 488 bp subsequence of mutant genes containing mutations D516V (GAC→GTC), *rpoB* H526D (CAC→GAC), H526Y (CAC→TAC), *rpoB* S531L (TCG→TTG), and WT were synthesized (Integrated DNA Technologies). The selected *M. tuberculosis* genes targeting INH and RIF resistance are commonly associated with MDR-TB cases [37,41,42,43].

The plasmids containing mutations of interest were linearized by restriction digestion for 120 min at 37 °C in 50 µL containing 10 units of restriction endonuclease *ClaI* with appropriate buffer supplied by the manufacturer (New England Biolabs, Ipswich, MA, USA). The reaction mix was incubated for 20 min at 65 °C to inactivate the enzyme, and the concentration of each linearized plasmid was determined in a NanoDrop instrument (Thermo Fisher Scientific, Inc., Waltham, MA, USA). The plasmids were diluted in nuclease-free water to create stock solutions containing known quantities of linearized target plasmids/µL.

The genomic DNA (gDNA) of INH- and RIF-resistant *M. tuberculosis* was provided by the Kreiswirth laboratory (CDI, Hackensack Meridian Health, Nutley, NJ, USA). The gDNA sequences were verified by DNA sequence analysis (Integrated DNA Technologies). WT H37Rv DNA was obtained from BEI resources (NIAID, NIH, Washington, DC, USA).

### 4.5. Preparation of DNA Samples

Template DNA for RT-PCR assays was prepared using pDNA/gDNA. A calculated number of mutant DNA copies was mixed with a calculated number of WT DNA copies to create varying ratios of mutant DNA against a WT background. Serial dilutions of mutant DNA (10^5^–1 copies) were mixed with 10^5^ or 10^4^ copies of WT DNA. Tested Mutant:WT ratios were 10^5^:10^5^, 10^4^:10^5^, 10^3^:10^5^, 10^2^:10^5^, 50:10^5^, 25:10^5^, 10:10^5^, 1:10^5^, 0:10^5^ and 10^5^:10^4^, 10^4^:10^4^, 10^3^:10^4^, 10^2^:10^4^, 50:10^4^, 25:10^4^, 10:10^4^, 1:10^4^, 0:10^4^. Mutant DNA copies 10^5^, 10^4^, 10^3^, 10^2^, 50, 25, 10 and 1 were used as control.

### 4.6. Assay Composition and Testing Procedure

Monoplex RT-PCR assays were performed with a SuperSelective forward primer and a conventional reverse primer on an AriaMx Real-Time PCR System (Agilent Technologies, Santa Clara, CA, USA). All amplifications were carried out in 0.2 mL clear tubes (Agilent Technologies, Santa Clara, CA, USA) in a final volume of 25 µL, containing 1X PCR buffer supplemented with 25 mM tetramethylammonium chloride (TMAC) (Sigma-Aldrich, St. Louis, MO, USA), 0.25% Tween20 (Sigma-Aldrich, St. Louis, MO, USA), 3 mM MgCl2, 0.05 U/µL Platinum Taq DNA polymerase (Thermo Fisher Scientific, Inc., Waltham, MA, USA), 0.25 mM dNTP mix, 0.1 µM each of forward and reverse primer, and 0.5X Sybr Green (Thermo Fisher Scientific, Inc., Waltham, MA, USA) to monitor template amplification. The reaction mixtures were incubated for 2 min at 95 °C to activate the Platinum Taq DNA polymerase, followed by 50–65 cycles of DNA denaturation at 95 °C for 15 s, primer annealing at 60 °C for 20 s, and extension at 72 °C for 20 s. Fluorescence was monitored and measured during the annealing step.

For the detection of mutants using molecular beacon probes in monoplex assays, asymmetric PCR conditions were optimized (Figures S3 and S4) to maximize the intensity of fluorescence signals. RT-PCR assay conditions were the same as described above, except that the forward and reverse primers were used at a 1:5 ratio, with final concentrations of 0.1 µM and 0.5 µM, respectively. This assay used 0.1 µM of molecular beacon probe instead of Sybr Green to monitor amplification.

Multiplex assays with molecular beacon probes were carried out in 0.2 mL white polypropylene PCR tubes (USA Scientific, Ocala, FL, USA) in a Bio-Rad CFX96 Real-Time System (Bio-Rad Laboratories, Hercules, CA, USA). RT-PCR assay conditions were the same as those used in the monoplex assays, except that 3 primer pairs and 3 probes were used (*katG*, *inhA*, *rpoB*) for multiplexing. The final concentration of each forward primer (*katG*, *inhA*, *rpoB*) was 0.1 µM, each reverse primer (*katG*, *inhA*, *rpoB*) 0.5 µM, probe *katG* 0.25 µM, probe *inhA* 0.1 µM, and probe *rpoB* 0.25 µM.

The RT-PCR assays were initially optimized on linearized plasmids containing a subsequence of the gene containing respective mutations as template DNA because of the availability of a large amount of material. The assay results were then confirmed by testing the optimized assay conditions on *M. tuberculosis* gDNA. RT-PCR conditions were tested for mutant DNA template at copy numbers 10^5^-1 in a background of 10^5^/10^4^ copies of WT DNA template. Mutant DNA at the copy numbers 10^5^-1 with no background of WT DNA was used as an assay control. Nuclease-free water was used as a no template control. Experiments were performed on duplicate samples and repeated at least 3 times.

### 4.7. Clinical Samples

Twenty-three *M. tuberculosis* DNA samples were isolated from archived sputum samples of anonymized TB patients with unknown drug resistance. These de-identified and anonymized sputum specimens were thrashed samples collected as part of routine TB diagnosis screening. Hence, no informed consent was required to extract bacterial DNA from the specimen, and the archived samples were accessed exclusively to rationally generate beneficial and scientifically valid assays to improve patient health. To isolate the gDNA, the sputum samples were first decontaminated and homogenized by using NALC-NaOH, then 2 mL of sputum was taken in an Oakridge tube, and an equal amount of 1% N-Acetyl-L-cysteine, 4% sodium hydroxide, and 2.9% sodium citrate was added. The suspension was incubated at 37 °C for 15 min and added with 45 mL of phosphate buffer, followed by centrifugation at 5000 rpm for 15 min. The obtained pellet was re-suspended in 1 mL of nuclease-free water. For DNA extraction, 1 mL of the decontaminated sample was spun again and re-suspended in 50 μL of Genolyse Lysis buffer (Hain Lifesciences GmbH, Hardwiesentrabe, Nehren, Germany), followed by heat-killing at 95 °C for 5 min to lyse the bacterial cells. Subsequently, the Genolyse Neutralizing buffer (50 μL) was added to the suspension, and the resulting DNA in the supernatant was pelleted by centrifugation at 13,000 rpm for 5 min.

## Figures and Tables

**Table 1:** Limit of detection (LoD) of mutant DNA from clinical isolates.

Mutation	SuperSelective Forward Primer	Conventional Reverse Primer	LoD *	No. of gDNA Samples Tested
*katG* (AGC→ACC)	20-14/14-5:1:0	*katG*_rev2	1	7
*katG* (AGC→ACA)	20-14/14-5:2:0	*katG*_rev2	1	2
*inhA* -8T→A	20-14/14-6:1:0	*inhA*_rev1	1	2
*inhA* -15C→T	18-14/13-7:1:0	*inhA*_rev1	1	4
*inhA* -17G→T	20-14/13-7:1:0	*inhA*_rev2	1	1
D516V (GAC→GTC)	20-14/13-7:1:0	*rpoB*_rev1	1	3
H526D (CAC→GAC)	20-14/13-7:1:0	*rpoB*_rev1	1	2
H526Y (CAC→TAC)	20-14/13-7:1:0	*rpoB*_rev1	1	2
S531L (TCG→TTG)	20-14/13-7:1:0	*rpoB*_rev1	1	4

* LoD: The lowest number of detectable copies of mutant DNA in a background of 10^4^ copies of WT DNA.

**Table 2:** Detection of mutations in clinical samples using SuperSelective primers.

Sample	Age/Sex	Total DNA (*rrs* Copies)	*katG* (AGC>ACC)		*inhA* (-15C→T)		*rpoB* (S531L)	
			Number of Mutant DNA Copies Detected by SuperSelective Primers	DNA Sequencing Result	Number of Mutant DNA Copies Detected by SuperSelective Primers	DNA Sequencing Result	Number of Mutant DNA Copies Detected by SuperSelective Primers	DNA Sequencing Result
AVR1	36/M	2595	2328	AGC > ACC	40	WT	2167	S531L
AVR2	28/M	254	5	WT	38	WT	284	S531L
AVR4	39/F	498	679	-	0	-	0	-
AVR7	33/M	540	1086	AGC > ACC	0	WT	1332	S531L
AVR8	49/M	979	601	-	0	-	0	-
AVR9	37/F	2867	2039	AGC > ACC	0	WT	2827	S531L
AVR11	62/F	24,693	2	WT	0	WT	4	WT
AVR12	28/F	3294	2262	AGC > ACC	0	WT	3992	S531L
AVR13	24/F	32	33	AGC > ACC	0	WT	42	S531L
AVR14	70/F	129	36	AGC > ACC	0	WT	57	S531L
AVR16	26/M	2038	534	AGC > ACC	0	WT	1184	S531L
AVR18	27/F	2235	0	-	84	-	0	-
AVR19	42/M	2680	0	-	0	-	0	-
AVR23	26/F	2867	0	-	0	-	0	-
AVR24	19/F	2434	0	-	0	-	0	-
AVR25	38/F	238	350	-	0	-	0	-
AVR26	18/F	197	197	AGC > ACC	0	WT	208	S531L
AVR27	26/F	2857	0	-	0	-	0	-
AVR30	28/M	1617	3885	-	0	-	0	-
AVS32	18/M	855	0	-	0	-	0	-
AVS34	42/F	3758	0	-	0	-	0	-
AVS35	57/M	5559	0	WT	10	WT	2	WT
AVS38	35/F	280	38	-	0	-	0	-

## Data Availability

All data generated and analyzed for this study are included in this article. All materials used and described in the methods are commercially available.

## Supplementary Materials

The following supporting information can be downloaded at: https://www.mdpi.com/article/10.3390/ijms232415752/s1
