# Supplementary material for: Ultrasensitive Detection of Multidrug-Resistant Mycobacterium tuberculosis Using SuperSelective Primer-Based Real-Time PCR Assays
Source: Int J Mol Sci. 2022 Dec 12;23(24):15752. doi: 10.3390/ijms232415752 (PMC9779475; doi:10.3390/ijms232415752)
Supplement: Supplementary file 1 [file ijms-23-15752-s001.zip › ijms-2010527-supplementary_REV.pdf]

Supplementary Data

Ultrasensitive Detection of Multidrug-Resistant *Mycobacterium tuberculosis* Using SuperSelective Primer-Based Real-Time PCR Assays

Anshika Narang [1], Salvatore A. E. Marras [1,*], Natalia Kurepina [2], Varsha Chauhan [3], Elena Shashkina [2], Barry Kreiswirth [2], Mandira Varma-Basil [3], Christopher Vinnard [4] and Selvakumar Subbian [1,*]

1 Public Health Research Institute, New Jersey Medical School, Rutgers University, Newark, NJ 07103, USA
2 Center for Discovery and Innovation, Nutley, NJ 07110, USA
3 Department of Microbiology, Vallabhbhai Patel Chest Institute, University of Delhi, Delhi 110021, India
4 New Jersey Medical School, Rutgers University, Newark, NJ 07103, USA
* Correspondence: marrassa@njms.rutgers.edu (S.A.E.M.); subbiase@njms.rutgers.edu (S.S.)

Citation: Narang, A.; Marras, S.A.E.; Kurepina, N.; Chauhan, V.; Shashkina, E.; Kreiswirth, B.; Varma-Basil, M.; Vinnard, C.; Subbian, S. Ultrasensitive Detection of Multidrug-Resistant *Mycobacterium tuberculosis* Using SuperSelective Primer-Based Real-Time PCR Assays. *Int. J. Mol. Sci.* **2022**, *23*, 15752. https://doi.org/10.3390/ijms232415752

Received: 19 October 2022
Accepted: 9 December 2022
Published: 12 December 2022

Publisher's Note: MDPI stays neutral with regard to jurisdictional claims in published maps and institutional affiliations.

Copyright: © 2022 by the authors. Submitted for possible open access publication under the terms and conditions of the Creative Commons Attribution (CC BY) license (https://creativecommons.org/licenses/by/4.0/).

a)

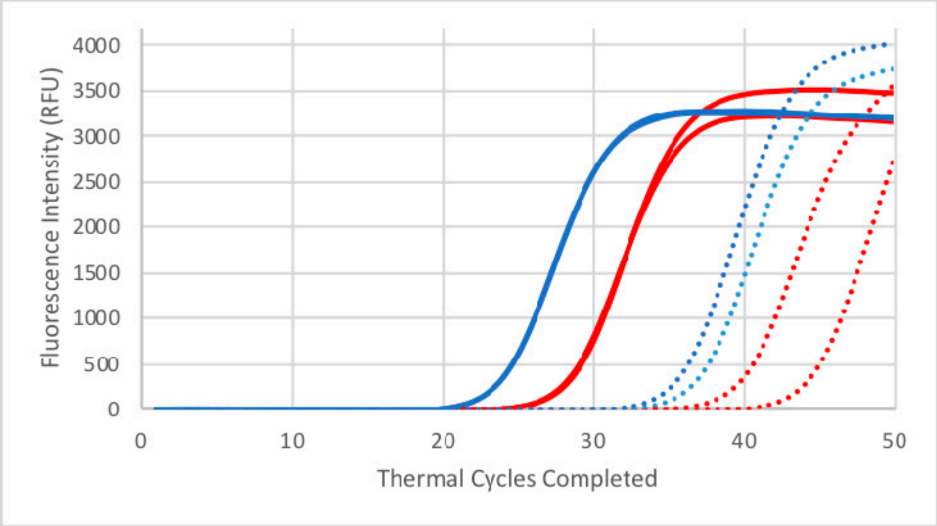

b)

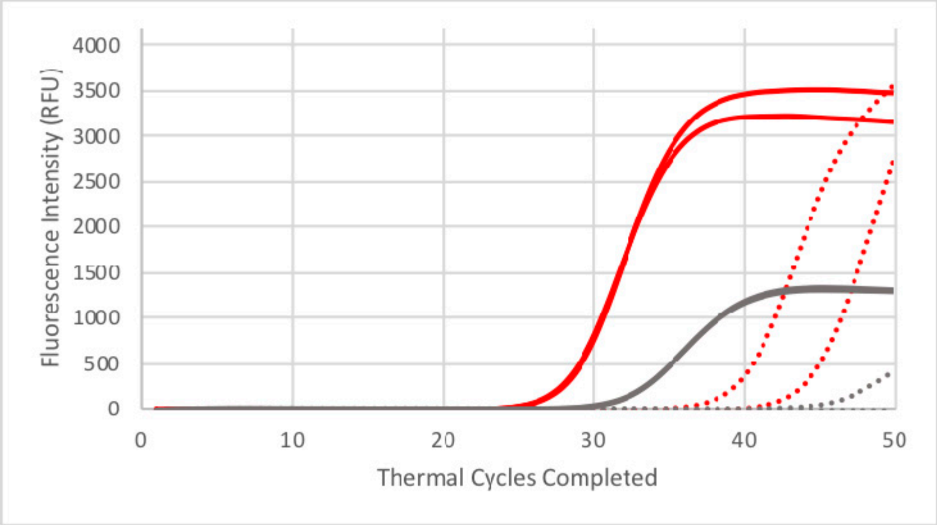

Figure S1. Effect of increase in length of the foot and intervening sequence of a SuperSelective primer on its performance. The plot shows the results of PCR assays for the detection of *inhA* promoter -15 mutation using 10^5 copies of mutant pDNA (solid lines) and 10^5 copies of WT pDNA (dotted lines). a. Comparison of threshold cycles with SuperSelective primer inh15_SSP3 (18-14/14-6:1:0) (red) and inh15_SSP4 (18-14/13-7:1:0) (blue) with foot length 7bp and 8bp respectively. b. Comparison of threshold cycles with SuperSelective primer inh15_SSP3 (18-14/14-6:1:0) (red) and inh15_SSP2 (18-14/21-6:1:0) (grey) with intervening sequence length 14bp and 21bp respectively.

a)

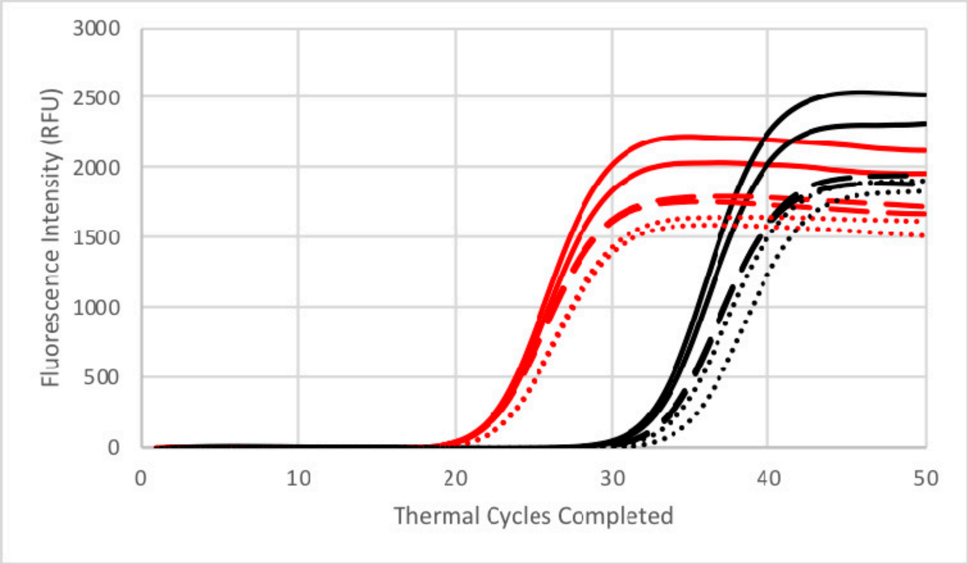

b)

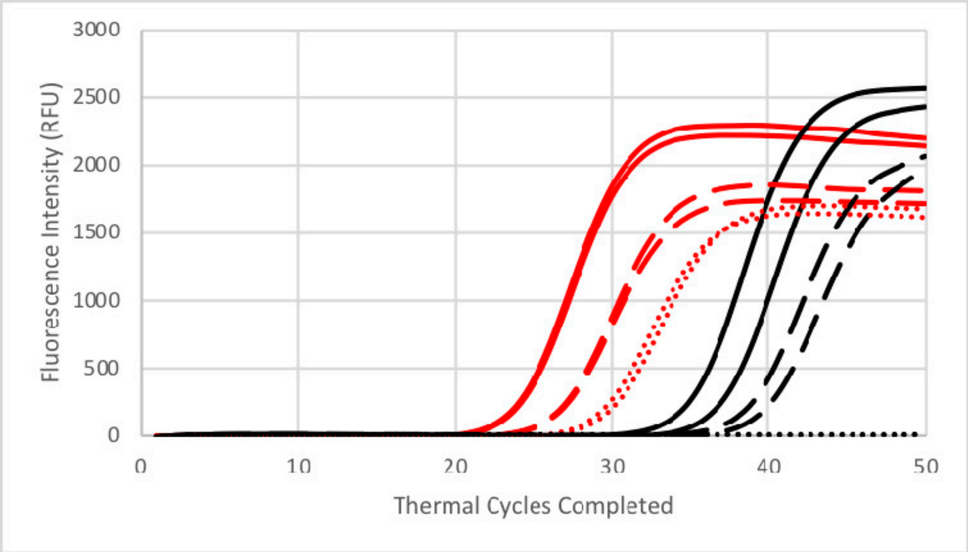

Figure S2. Efficiency of SuperSelective primer designs with symmetric and asymmetric bubble under different annealing temperatures. a. inh15_SSP8 (18-8/8-7:1:0) – symmetric bubble, b. inh15_SSP7 (18-20/8-7:1:0) – asymmetric bubble. The plots show the results of PCR assays carried out for the detection of *inhA* promoter -15 mutation using 10^5 copies of mutant pDNA (red) and 10^5 copies of WT pDNA (black) at an annealing temperature of 60°C (Solid lines), 64°C (Broken lines), and 66°C (Dotted lines).

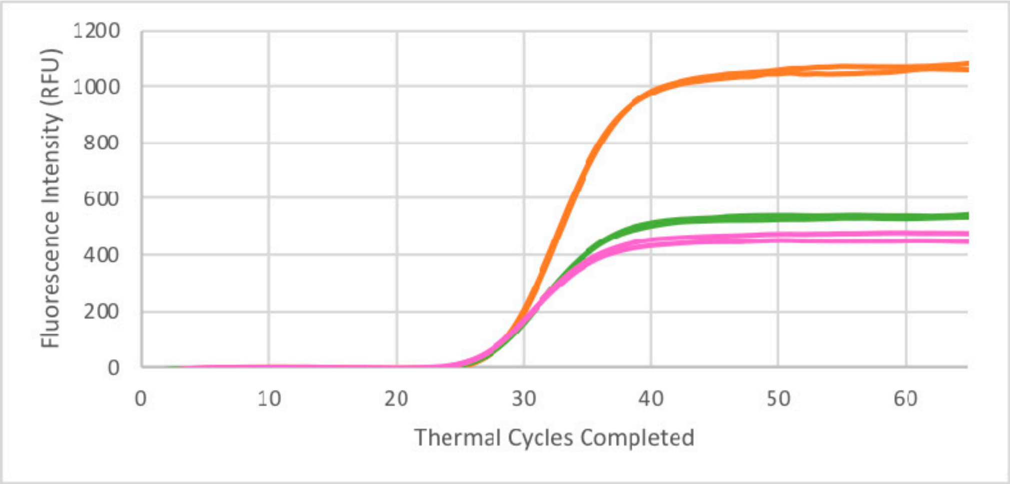

Figure S3. Fluorescence signal intensity of *inhA* probe at different molecular beacon concentrations. The plot shows amplification of 10^5 copies of mutant target DNA template using SuperSelective primer inh8_SSP1 (18-14/14-6:1:0) and a conventional reverse primer for the detection of *inhA* promoter mutation -8T-->A at molecular beacon probe concentrations 0.25µM (orange), 0.125µM (green) and 0.1µM (pink). There was no significant difference between the signal intensity with 0.1µM and 0.125µM probe; however, 0.25µM probe resulted in signals with the highest fluorescence intensity.

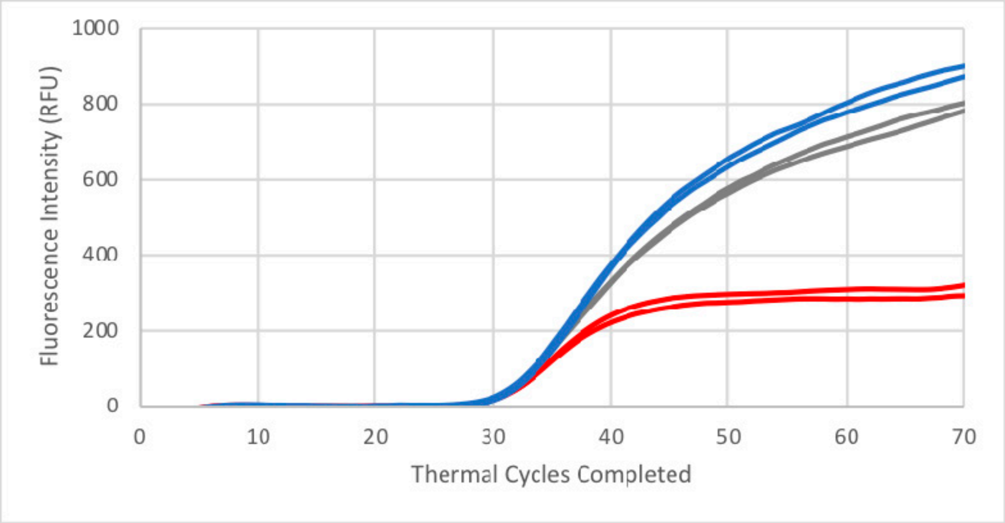

Figure S4. Fluorescence signal intensity of *rpoB* probe in asymmetric PCR. The plot shows the amplification of 10^5 copies of *rpoB* D516V GAC-->GTC mutant template with SuperSelective primer rpoB516_SSP4 (18-14/13-7:1:0) and a conventional reverse primer in the ratio 1:1, 1:5 and 1:10. Asymmetric PCR with forward primer 0.1µM, reverse primer 0.5µM (grey) and 1µM (blue) resulted in higher fluorescence intensity as compared to symmetric PCR with forward and reverse primer 0.1µM (red). However, there was no significant difference in fluorescence intensity with reverse primer 0.5µM and 1µM.

Table S1: RT-PCR assay conditions tested for the optimization of SuperSelective primers for the detection of *inhA* promoter mutation -15C-->T.

Forward Primer	Reverse Primer	Annealing Temperature (°C)	TMAC (mM)*
inh15_SSP2 (18-14/21-6:1:0)	inhA_rev1	60	25
inh15_SSP3 (18-14/14-6:1:0)	inhA_rev1	60, 62	25
inh15_SSP3 (18-14/14-6:1:0)	inhA_rev2	60	25
inh15_SSP4 (18-14/13-7:1:0)	inhA_rev1	60, 62, 66	25
inh15_SSP4 (18-14/13-7:1:0)	inhA_rev3, inhA_rev4	60	25
inh15_SSP5 (18-15/13-7:1:0)<br>inh15_SSP6 (18-17/13-7:1:0)	inhA_rev1	60, 62	25
inh15_SSP7 (18-20/8-7:1:0)<br>inh15_SSP8 (18-8/8-7:1:0)<br>inh15_SSP9 (18-8/20-7:1:0)	inhA_rev1	60, 64, 66	25
inhA_for2<br>inh15_SSP4 (18-14/13-7:1:0)	SSP10 (18-14/14-6:1:0)	60	25
inh15_SSP4 (18-14/13-7:1:0)<br>inh15_SSP7 (18-20/8-7:1:0)	SSP10 (18-14/14-6:1:0)	60, 64	0, 25
inhA_for2<br>inhA_for4<br>inh15_SSP4 (18-14/13-7:1:0)<br>inh15_SSP7 (18-20/8-7:1:0)	SSP11 (18-14/13-7:1:0)	60	0, 25
inhA_for2<br>inhA_for4<br>inh15_SSP4 (18-14/13-7:1:0)<br>inh15_SSP7 (18-20/8-7:1:0)	SSP12 (18-14/8-6:1:0)<br>SSP13 (18-14/8-7:1:0)	60	0
inh15_SSP4 (18-14/13-7:1:0)<br>inh15_SSP7 (18-20/8-7:1:0)	SSP10 (18-14/14-6:1:0)<br>SSP11 (18-14/13-7:1:0)<br>SSP12 (18-14/8-6:1:0)<br>SSP13 (18-14/8-7:1:0)	64, 66	0
inh15_SSP4 (18-14/13-7:1:0)	SSP17,18	60	25
inh15_SSP22	SSP10 (18-14/14-6:1:0)	60	0
inh15_SSP14-16, 19-21	inhA_rev1	60	25
inh15_SSP23-25	SSP10 (18-14/14-6:1:0)	60	25
inh15_SSP3 (18-14/14-6:1:0)<br>inh15_SSP4 (18-14/13-7:1:0)<br>inh15_SSP7 (18-20/8-7:1:0)	inhA_rev1	60	0

*Addition of TMAC was always accompanied by Tween 20 (0.25%)

Table S2: List of primers used for *katG*, *inhA* and *rpoB* mutations.

Gene	Mutation	Primer ID	Strand +/-	Design	Sequence
katG	S315T AGC-->ACA	katG315T2_SSP1	+	20-14/14-5:2:0	<u>GCTCGTATGGCACCGGAACC</u>*ACACTACCGCGACC***CACCACA**
katG	S315T AGC-->ACC	katG315T1_SSP1	+	20-14/14-5:1:0	<u>GCTCGTATGGCACCGGAACC</u>*ACACTACCGCGACC***CACCAC**
katG	S315T AGC-->ACC	katG315T1_SSP2	+	20-14/13-6:1:0	<u>GCTCGTATGGCACCGGAACC</u>*TCACTACAGAAACC***TCACCAC**
katG		katG_rev1	-		ATTTCGTCGGGGTGTTCGTC
katG		katG_rev2	-		TCGTAGCCGTACAGGATCTCG
inhA	-8T-->A	inh8_SSP1	-	18-14/14-6:1:0	<u>GTTTGGCCCCTTCAGTGG</u>*AATATTGTTGATAA***CCGACAT**
inhA	-8T-->A	inh8_SSP2	-	20-14/14-6:1:0	<u>GGGTTTGGCCCCTTCAGTGG*AATATTGTTGATAA*</u>**CCGACAT**
inhA	-15C-->T	inh15_SSP2	-	18-14/21-6:1:0	<u>GTTTGGCCCCTTCAGTGG</u>*AATATTGTCATGTT***CCTATCA**
inhA	-15C-->T	inh15_SSP3	-	18-14/14-6:1:0	<u>CCCTTCAGTGGCTGTGGC</u>*TATATTGTCATGTT***CCTATCA**
inhA	-15C-->T	inh15_SSP4	-	18-14/13-7:1:0	<u>CCCTTCAGTGGCTGTGGC</u>*TATATTGTCATGTT***ACCTATCA**
inhA	-15C-->T	inh15_SSP5	-	18-15/13-7:1:0	<u>CCCTTCAGTGGCTGTGGC</u>*TATATTAGTCATGTT***ACCTATCA**
inhA	-15C-->T	inh15_SSP6	-	18-17/13-7:1:0	<u>CCCTTCAGTGGCTGTGGC</u>*TATATTATCGTCATGTT***ACCTATCA**
inhA	-15C-->T	inh15_SSP7	-	18-20/8-7:1:0	<u>CAGTGGCTGTGGCAGTCA</u>*TCTAGTACACTCGTCATGTT***ACCTATCA**
inhA	-15C-->T	inh15_SSP8	-	18-8/8-7:1:0	<u>CAGTGGCTGTGGCAGTCA</u>*GTCATGTT***ACCTATCA**
inhA	-15C-->T	inh15_SSP9	-	18-8/20-7:1:0	<u>GTTTGGCCCCTTCAGTGG</u>*ATCATGTT***ACCTATCA**
inhA	-15C-->T	inh15_SSP10	+	18-14/14-6:1:0	<u>CTCGTGGACATACCGATT</u>*GTTGTTCATCAGTA***GCGAGAT**
inhA	-15C-->T	inh15_SSP11	+	18-14/13-7:1:0	<u>CTCGTGGACATACCGATT</u>*GTTGTTCATCAGT***GGCGAGAT**
inhA	-15C-->T	inh15_SSP12	+	18-14/8-6:1:0	<u>CTCGTGGACATACCGATT</u>*GTTGTTCA***GCGAGAT**
inhA	-15C-->T	inh15_SSP13	+	18-14/8-7:1:0	<u>CTCGTGGACATACCGATT</u>*GTTG*

					*TTCA***GGCGAGAT**
inhA	-15C-->T	inh15_SSP14	-	18-14/14-6:1:0	CCCTTCAGTGGCTGTGGC*TATATTGTCATGTT***CCTCTCA**
inhA	-15C-->T	inh15_SSP15	-	18-14/13-7:1:0	CCCTTCAGTGGCTGTGGC*TATATTGTCATGTT***ACCTCTCA**
inhA	-15C-->T	inh15_SSP16	-	18-20/8-7:1:0	CAGTGGCTGTGGCAGTCA*TCTAGTACACTCGTCATGTT***ACCTCTCA**
inhA	-15C-->T	inh15_SSP17	+	20-14/14-6:1:0	CGCTCGTGGACATACCGATT*GTTGTTCATCAGTA***GCGAGAT**
inhA	-15C-->T	inh15_SSP18	+	20-14/13-7:1:0	CGCTCGTGGACATACCGATT*GTTGTTCATCAGT***GGCGAGAT**
inhA	-15C-->T	inh15_SSP19	-	18-14/14-6:1:2	CCCTTCAGTGGCTGTGGC*TATATTGTCATGTT***CCTATCATC**
inhA	-15C-->T	inh15_SSP20	-	18-14/13-7:1:2	CCCTTCAGTGGCTGTGGC*TATATTGTCATGTT***ACCTATCATC**
inhA	-15C-->T	inh15_SSP21	-	18-20/8-7:1:2	CAGTGGCTGTGGCAGTCA*TCTAGTACACTCGTCATGTT***ACCTATCATC**
inhA	-15C-->T	inh15_SSP22	-	18-14/14-3:1:2	TTCAGTGGCTGTGGCAGT*ATTGTTACATTATA***ATCATC**
inhA	-15C-->T	inh15_SSP23	-	18-14/14-3:2:1	TTCAGTGGCTGTGGCAGT*ATTGTTACATTATA***ATCACC**
inhA	-15C-->T	inh15_SSP24	-	18-14/14-6:2:1	CCCTTCAGTGGCTGTGGC*TATATTGTCATGTT***CCTATCACC**
inhA	-15C-->T	inh15_SSP25	-	18-14/14-6:2:0	CCCTTCAGTGGCTGTGGC*TATATTGTCATGTT***CCTATCAC**
inhA	-15C-->T	inh15_SSP26	-	18-10/14-6:1:0	CCCTTCAGTGGCTGTGGC*TATATTGTCATGTT***CCTATCA**
inhA	-15C-->T	inh15_SSP27	-	20-14/13-7:1:0	GCCCCTTCAGTGGCTGTGGC*TATATTGTCATGTT***ACCTATCA**
inhA	-15C-->T	inh15_SSP28	-	18-14/12-8:1:0	CCCTTCAGTGGCTGTGGC*TATATTGTCATGTT***AACCTATCA**
inhA	-17G-->T	inh17_SSP1	-	18-14/15-6:1:0	CCTTCAGTGGCTGTGGCA*TATATTGTCATGTT***TATCGTA**
inhA	-17G-->T	inh17_SSP2	-	18-14/14-6:1:0	CTTCAGTGGCTGTGGCAG*TATATTGTCATGTT***TATCGTA**
inhA	-17G-->T	inh17_SSP3	-	18-14/13-7:1:0	CTTCAGTGGCTGTGGCAG*TATATTGTCATGTT***CTATCGTA**
inhA	-17G-->T	inh17_SSP4	-	20-14/14-6:1:0	CCCTTCAGTGGCTGTGGCAG*TATATTGTCATGTT***TATCGTA**

inhA	-17G-->T	inh17_SSP5	-	20-14/13-7:1:0	CCCTTCAGTGGCTGTGGCAG_TATATTGTCATGTT_**CTATCGTA**
inhA		inhA_rev1	+		GGAAATCGCAGCCACGTTAC
inhA		inhA_rev2	+		TTACGCTCGTGGACATACCG
inhA		inhA_rev6	+		CGTAACCCCAGTGCGAAAGT
inhA		inhA_for2	-		CTCGTGGACATACCGATT
inhA		inhA_for4	-		CACGTTACGCTCGTGGACAT
rpoB	D516V (GAC-->GTC)	rpoB516_SSP1	-	20-14/14-6:1:0	TCGGCGCTTGTGGGTCAACC_TGCTTGTATACACA_**TTCTGGA**
rpoB	D516V (GAC-->GTC)	rpoB516_SSP2	-	18-14/14-6:1:0	GGCGCTTGTGGGTCAACC_TGCTTGTATACACA_**TTCTGGA**
rpoB	D516V (GAC-->GTC)	rpoB516_SSP3	-	20-14/13-7:1:0	TCGGCGCTTGTGGGTCAACC_TGCTTGTATACACA_**GTTCTGGA**
rpoB	D516V (GAC-->GTC)	rpoB516_SSP4	-	18-14/13-7:1:0	GGCGCTTGTGGGTCAACC_TGCTTGTATACACA_**GTTCTGGA**
rpoB	H526D (CAC-->GAC)	rpoB526D_SSP1	-	20-14/14-6:1:0	GACAGACCGCCGGGCCCCAG_TATTACAGTAAATT_**GCTTGTC**
rpoB	H526D (CAC-->GAC)	rpoB526D_SSP2	-	16-14/22-6:1:0	ACGTGACAGACCGCCG_TATTACAGTAAATT_**GCTTGTC**
rpoB	H526D (CAC-->GAC)	rpoB526D_SSP3	-	16-14/21-7:1:0	ACGTGACAGACCGCCG_TATTACAGTAAATT_**CGCTTGTC**
rpoB	H526D (CAC-->GAC)	rpoB526D_SSP4	-	18-14/14-6:1:0	CAGACCGCCGGGCCCCAG_TATTACAGTAAATT_**GCTTGTC**
rpoB	H526D (CAC-->GAC)	rpoB526D_SSP5	-	18-14/22-6:1:0	TCACGTGACAGACCGCCG_TATTACAGTAAATT_**GCTTGTC**
rpoB	H526D (CAC-->GAC)	rpoB526D_SSP6	-	20-14/13-7:1:0	GACAGACCGCCGGGCCCCAG_TATTACAGTAAATT_**CGCTTGTC**
rpoB	H526D (CAC-->GAC)	rpoB526D_SSP7	-	18-14/13-7:1:0	CAGACCGCCGGGCCCCAG_TATTACAGTAAATT_**CGCTTGTC**
rpoB	H526D (CAC-->GAC)	rpoB526D_SSP8	-	18-14/21-7:1:0	TCACGTGACAGACCGCCG_TATTACAGTAAATT_**CGCTTGTC**
rpoB	H526Y (CAC-->TAC)	rpoB526Y_SSP1	-	20-14/14-6:1:0	GACAGACCGCCGGGCCCCAG_TATTACAGTAAATT_**GCTTGTA**
rpoB	H526Y (CAC-->TAC)	rpoB526Y_SSP2	-	16-14/22-6:1:0	ACGTGACAGACCGCCG_TATTACAGTAAATT_**GCTTGTA**
rpoB	H526Y (CAC-->TAC)	rpoB526Y_SSP3	-	16-14/21-7:1:0	ACGTGACAGACCGCCG_TATTACAGTAAATT_**CGCTTGTA**
rpoB	H526Y (CAC-->TAC)	rpoB526Y_SSP4	-	18-14/14-6:1:0	CAGACCGCCGGGCCCCAG_TATTACAGTAAATT_**GCTTGTA**
rpoB	H526Y	rpoB526Y_SSP5	-	18-14/22-6:1:0	TCACGTGACAGACCGCCG_TATT_

	(CAC-->TAC)				*ACAGTAAATT***GCTTGTA**
rpoB	H526Y (CAC-->TAC)	rpoB526Y_SSP6	-	20-14/13-7:1:0	GACAGACCGCCGGGCCCCAG*TATTACAGTAAATT***CGCTTGTA**
rpoB	H526Y (CAC-->TAC)	rpoB526Y_SSP7	-	18-14/13-7:1:0	CAGACCGCCGGGCCCCAG*TATTACAGTAAATT***CGCTTGTA**
rpoB	H526Y (CAC-->TAC)	rpoB526Y_SSP8	-	18-14/21-7:1:0	TCACGTGACAGACCGCCG*TATTACAGTAAATT***CGCTTGTA**
rpoB	S531L (TCG-->TTG)	rpoB531_SSP1	-	20-14/14-6:1:0	CCCGGCACGCTCACGTGACA*TGTTATACTAAATT***AGCGCCA**
rpoB	S531L (TCG-->TTG)	rpoB531_SSP2	-	20-14/12-6:1:0	CGGCACGCTCACGTGACAGA*TGTTATACTAAATT***AGCGCCA**
rpoB	S531L (TCG-->TTG)	rpoB531_SSP3	-	18-14/14-6:1:0	CGGCACGCTCACGTGACA*TGTTATACTAAATT***AGCGCCA**
rpoB	S531L (TCG-->TTG)	rpoB531_SSP4	-	20-14/13-7:1:0	CCCGGCACGCTCACGTGACA*TGTTATACTAAATT***CAGCGCCA**
rpoB	S531L (TCG-->TTG)	rpoB531_SSP5	-	20-14/11-7:1:0	CGGCACGCTCACGTGACAGA*TGTTATACTAAATT***CAGCGCCA**
rpoB	S531L (TCG-->TTG)	rpoB531_SSP6	-	18-14/13-7:1:0	CGGCACGCTCACGTGACA*TGTTATACTAAATT***CAGCGCCA**
rpoB	S531L (TCG-->TTG)	rpoB_rev1	+		GAGGCGATCACACCGCAGAC
rpoB	S531L (TCG-->TTG)	rpoB_rev2	+		GCCGCGATCAAGGAGTTCTTC
